# Differences in the superspreading potentials of COVID-19 across contact settings

**DOI:** 10.1186/s12879-022-07928-9

**Published:** 2022-12-12

**Authors:** Yanji Zhao, Shi Zhao, Zihao Guo, Ziyue Yuan, Jinjun Ran, Lan Wu, Lin Yu, Hujiaojiao Li, Yu Shi, Daihai He

**Affiliations:** 1 Department of Applied Mathematics, Hong Kong Polytechnic University, Hong Kong, China; 2 JC School of Public Health and Primary Care, Chinese University of Hong Kong, Hong Kong, China; 3 CUHK Shenzhen Research Institute, Shenzhen, China; 4 Centre for Health Systems and Policy Research, Faculty of Medicine, Chinese University of Hong Kong, Hong Kong, China; 5 Department of Civil and Environmental Engineering, Hong Kong Polytechnic University, Hong Kong, China; 6 School of Public Health, Shanghai Jiao Tong University School of Medicine, Shanghai, China; 7 Department of Mathematics and Physics, Xi’an Jiaotong-Liverpool University, Suzhou, China; 8 Faculty of Arts and Sciences, University of Toronto, Toronto, Canada; 9 Yale School of Management, Yale University, New Haven, USA; 10 Research Institute for Future Food, Hong Kong Polytechnic University, Hong Kong, China

**Keywords:** COVID-19, Contact settings, Superspreading, Transmission heterogeneity

## Abstract

**Background:**

Superspreading events (SSEs) played a critical role in fueling the COVID-19 outbreaks. Although it is well-known that COVID-19 epidemics exhibited substantial superspreading potential, little is known about the risk of observing SSEs in different contact settings. In this study, we aimed to assess the potential of superspreading in different contact settings in Japan.

**Method:**

Transmission cluster data from Japan was collected between January and July 2020. Infector-infectee transmission pairs were constructed based on the contact tracing history. We fitted the data to negative binomial models to estimate the effective reproduction number (*R*) and dispersion parameter (*k*). Other epidemiological issues relating to the superspreading potential were also calculated.

**Results:**

The overall estimated *R* and *k* are 0.561 (95% CrI: 0.496, 0.640) and 0.221 (95% CrI: 0.186, 0.262), respectively. The transmission in community, healthcare facilities and school manifest relatively higher superspreading potentials, compared to other contact settings. We inferred that 13.14% (95% CrI: 11.55%, 14.87%) of the most infectious cases generated 80% of the total transmission events. The probabilities of observing superspreading events for entire population and community, household, health care facilities, school, workplace contact settings are 1.75% (95% CrI: 1.57%, 1.99%), 0.49% (95% CrI: 0.22%, 1.18%), 0.07% (95% CrI: 0.06%, 0.08%), 0.67% (95% CrI: 0.31%, 1.21%), 0.33% (95% CrI: 0.13%, 0.94%), 0.32% (95% CrI: 0.21%, 0.60%), respectively.

**Conclusion:**

The different potentials of superspreading in contact settings highlighted the need to continuously monitoring the transmissibility accompanied with the dispersion parameter, to timely identify high risk settings favoring the occurrence of SSEs.

**Supplementary Information:**

The online version contains supplementary material available at 10.1186/s12879-022-07928-9.

## Introduction

During the past few years, the coronavirus disease 2019 (COVID-19) that caused by the severe acute respiratory syndrome coronavirus 2 (SARS-CoV-2) has been continuously spreading worldwide, posing a significant threat to public health. A comprehensive understanding on the epidemiological characteristics of COVID-19 underlies the strategic development of region-wide control policies to combat the epidemics. The fundamental biological parameters—basic reproduction number (*R*_0_) and effective reproduction number (*R*) describe the transmission potential of a typical infectious disease agent, that is, the average number of secondary cases generated by an infectious person in a completely and not completely susceptible population, respectively [1]. While for the COVID-19 epidemics, the differences arose in infectiousness, behavioral patterns and locally implemented public health interventions give rise to heterogeneous individual transmissibility [2, 3], which cannot be reflected by a single measurement of *R*_0_ [4].

A superspreading event (SSE) is defined as a transmission event involving an unusual large number of cases, initiated by the super-spreader. The SSE represented a heterogeneous transmission pattern, where the majority of the cases were seeded by a small fraction of super-spreaders [5, 6]. Herein, the aggregation of transmission for some superspreading cases has also drawn researchers’ attention, defined as “20/80” rule [5] in epidemiology, which implies that approximately 80% secondary infected cases and transmissions result from roughly 20% of primary cases. As a distinct feature of the transmission dynamics of COVID-19, SSEs played essential roles in aggravating the COVID-19 epidemics. For instance, in early November 2021 in Hong Kong, an outbreak in the community was caused by a few SSEs in entertainment places, which led to a major epidemic wave in the whole city [7]. In South Korea, the SSE seeded by the SARS-CoV-2 Omicron variants occurred in churches and schools, causing the disease to spread widely in the local community [8]. Characterizing the superspreading potential of the epidemics in the context could give policymakers a hint on how to effectively curb the local transmissions [9]. For example, identifying and shutting down the hot-spot contact settings favoring the occurrence SSE (e.g., bars, social parties, and gyms) could timely chop the transmission chains and prevent future large outbreaks. However, spurred by the increasing burden of spread of COVID-19, few researches have been involved in the potential of superspreading events in different contact setting.

As a forceful circumstantial evidence of community transmission and SSEs, Furuse et al. exemplified demographic information regarding some clusters of COVID-19 infectors and schematized their features in transmission chains from January to July 2020 in Japan with different contact settings of SSEs [10]. This study sought to explore the estimated effective reproductive numbers and dispersion parameters in offspring distributions based on the rearranged contact tracing data in transmission chains in Japan from [10]. Herein, with transmission clusters data collected during the early phase of the epidemics, we aimed to quantify the transmission risk and contrast of superspreading potential of the COVID-19 among different contact settings.

## Methods

We obtained data on 28 circumstances of transmission clusters from January to July 2020 in Japan [10]. Based on the contact tracing and exposure history of each case within the transmission clusters, we constructed infectee-infector transmission pairs. We thereafter extracted the number of secondary cases (i.e., infectees) that were directly generated by each infector for further analysis. We excluded the cases that are indirectly linked with the infectors. The identified transmission pairs were further grouped by different contact settings (i.e., community, health care facility, school, household, and workplace) according to where the transmission occurred. Specifically, the contact setting “community” represented the aggregation of transmission dynamics in scenarios of social parties, restaurants, bars, clubs, ceremonies, gyms, etc. Segmentation of subgroups in the contact setting “community” was not feasible since the counts of them were trivial and not statistically significant. Furthermore, those without detailed information regarding contact settings were also omitted.

To quantify the superspreading potential, we assumed the number of secondary cases seeded by each infector following a Negative binomial distribution [6], which was parameterized by an effective reproduction number (*R*) as the mean and a dispersion parameter (*k*). The *k* captured the heterogeneity in the individual transmissibility. A lower value of *k* indicated a higher transmission heterogeneity, and thereby a higher superspreading potential. The number of offspring cases generated by each seed case was fitted to a negative binomial model. For the model parameter estimation, Markov chain Monte Carlo (MCMC) method was applied to estimate the joint posterior distribution of *R* and *k*.

The proportion of the most infectious cases that seeded 80% of the total transmissions was calculated [11]. The probability that a seed case generates a cluster with size 10 or more and the probability of observing SSEs were also computed. In addition to incorporating the expected proportion of infectors generating at least one infected individual and the probability that a seed case generates a cluster with size 10 or more, some intuitive concepts, such as the proportion of the most infectious infectors responsible for 80% of infectees and the expected probability of superspreading events, were also attained based on estimated [12–14]. Followed by previous work [6], we defined the threshold of SSEs as the 99-th percentile of the Poisson distribution with the rate at reproduction number (Additional file 1). Any transmission event that is seeded by a single infector would be counted as an SSE if the number of secondary cases exceeds the threshold. We thereafter calculated the probability of observing SSEs seeded by a single infector according to the SSE threshold. Subgroup analysis in different contact settings was also conducted in the same procedure to obtain the above estimates. 95% credible intervals (CrI) for each estimate were calculated as well. Technical details of the methodology can be found in Additional file 1.

## Results

A total of 500 transmission pairs were constructed from the reported 28 transmission clusters. Of the settings where the identified transmission pairs occur, 31.1%, 25.6%, 28.7%, 4.0%, and 10.6% belonged to the community, household, health care facility, school and workplace, respectively. Among 1017 identified infectors, 75.0% of them led to no secondary cases, and 0.8% of them directly generated more than 10 cases. From the observed secondary case distribution and fitted negative binomial models, we estimated that the overall *R* and *k* were 0.561 (95% CrI: 0.496, 0.640) and 0.221 (95% CrI: 0.186, 0.262), respectively (Table 1).

**Table 1:** Summary of the estimated metrics of superspreading potentials under different contact settings

	Total	Community	Household	Health care facilities	School	Workplace
Reproduction number (*R*)	0.561 (0.496, 0.640)	0.107 (0.046, 0.331)	0.137 (0.110, 0.168)	0.186 (0.079, 0.409)	0.088 (0.028, 0.295)	0.080 (0.052, 0.138)
Dispersion parameter (*k*)	0.221 (0.186, 0.262)	0.004 (0.002, 0.007)	0.141 (0.098, 0.210)	0.004 (0.002, 0.006)	0.002 (0.001, 0.005)	0.019 (0.012, 0.029)
Probability of 1 infector generating ≥ 1 infectees	24.37% (21.47, 27.68)	1.32% (0.63, 2.68)	9.13% (7.11, 11.61)	1.53% (0.74, 2.51)	0.76% (0.34, 2.03)	3.09% (1.99, 4.95)
Proportion of infector seeding 80% transmission	13.14% (11.55, 14.87)	0.44% (0.21, 0.76)	6.39% (4.91, 8.24)	0.44% (0.22, 0.66)	0.22% (0.11, 0.55)	1.54% (0.99, 2.43)
Probability of observing SSEs	1.75% (1.57, 1.99)	0.49% (0.22, 1.18)	0.07% (0.06, 0.08)	0.67% (0.31, 1.21)	0.33% (0.13, 0.94)	0.32% (0.21, 0.60)
Probability of cluster size ≥ 10 seeded by 1 infector	3.87% (2.94, 5.24)	0.37% (0.16, 1.00)	0.05% (0.04, 0.06)	0.55% (0.24, 1.04)	0.26% (0.09, 0.80)	0.17% (0.10, 0.38)

The metrics were summarized as ‘median estimate (95% CrI)’ format

**Fig. 1:**
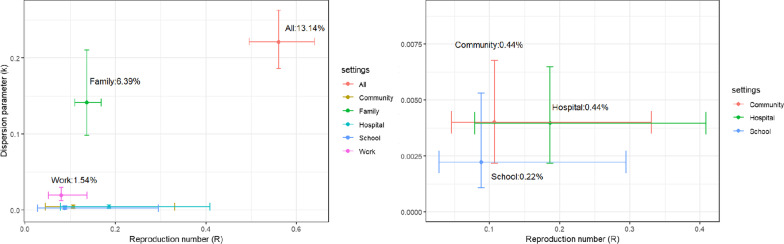
The Joint estimates of reproduction numbers and dispersion parameters of 6 settings (left) and three of them (right) specified of COVID-19. The two-dimension points are estimates of reproduction numbers and dispersion parameters. Vertical and horizonal lines indicate for each point are 95% credible intervals of reproduction numbers and dispersion parameters, respectively. The proportions of infector accounting for 80% of transmissions for each contact setting are indicated

Figure 1 illustrated the joint estimates of reproduction numbers and dispersion parameters in different contact settings with 95% credible intervals. Based on the estimated *R* value, the threshold of SSEs was determined to be 6, and there were 17 out of 500 (3.4%) transmission events identified as SSEs. We inferred that 80% of total transmissions were generated by 13.14% (95% CrI: 11.55%, 14.87%) of the most infectious seed cases.

Across all contact settings, the health care facility and household had a higher risk of transmission (larger value of *R*) whereas school, health care facility, and community had a higher superspreading potential (smaller value of *k*). The probability that an infector generates at least one secondary case was 24.37% (95% CrI: 21.47, 27.68). Furthermore, the probability of observing SSEs with a predefined threshold is 1.75% (95% CrI: 1.57, 1.99), and the probability that a seed case generates a transmission cluster with a size of 10 or greater is 3.87% (95% CrI: 2.94, 5.24). Other epidemiological results for mentioned contact settings are shown in Table 1.

## Discussion

Characterizing the superspreading potential could provide a better understanding of the transmission potential of the COVID-19 pandemic and help to formulate targeted public health interventions. In this study, using transmission cluster data collected during the early phase of the epidemic in Japan, we assessed the superspreading potential of COVID-19 within different contact settings.

The effective reproduction number for each contact setting and the whole population are all less than 1. It’s compatible with the scenario that the pandemic from January to July 2020 in Japan has been controlled with valid interventions before the new wave of counterattack and variants of the virus. The reproduction number of transmissions among hospitals was relatively higher than others, and the dispersion parameters of hospitals and schools were small, consistent with the scenario that there were more vulnerable individuals or higher risk of contact of cases in hospitals, healthcare facilities and schools. It was also concluded in [10] that rare superspreading events in community resulted from infectors from hospitals, healthcare facilities or schools, whereas some cases in hospitals, healthcare facilities or schools were caused by the transmission chains originating from community superspreading events, which may lead to low dispersion parameter in the distribution of offspring from communities.

We found that the early epidemics in Japan exhibited a significant superspreading potential (*k* = 0.22), which is in line with another study conducted during a similar study period (*k* = 0.23) [15], but is smaller than an estimate obtained in Hong Kong (*k* = 0.43) [16]. This discrepancy could be attributed to the differences in imposed control policies. In Japan, cluster-based measures that focused on identifying and preventing transmission clusters were adopted to curb the epidemics [17]. On the other hand, a series of social distancing interventions, including school closure, work-from-home-policy, and cancellation of mass gatherings, were implemented in Hong Kong [18], which may have a greater effect on reducing the potential of societal SSEs [19] and thus resulting in a relatively higher *k*. It was also concluded in [10] that rare superspreading events in community resulted from infectors from hospitals, healthcare facilities or schools, whereas some cases in hospitals, healthcare facilities or schools were caused by the transmission chains originated from community superspreading events, which may lead to a low dispersion parameter in the distribution of offspring from communities. The selection of threshold of superspreading events also vacillates the assessment of superspreading potential [20], as we defined the threshold of SSE for the COVID-19 as the 99-th percentile of the Poisson distribution of the basic reproduction number (*R*_0_). Meanwhile, the super-aged society in Japan [10] can also be deemed as the underlying cause of the estimates in each setting.

We also found that the risk of transmission and superspreading potentials varied across different contact settings. The higher estimated superspreading potential in schools and communities is consistent with a study conducted in South Korea, whereby the transmission chains in communities and schools were more heterogeneous (smaller *k*) than that in the household [21]. Besides Hong Kong and South Korea, compared to other contact settings, relatively more significant superspreading potential occurred in communities in some other regions since there has been a high likelihood of community gathering due to religions and folk custom, such as Kumbh Mela during April and May in India and Songkran festival in Thailand [22, 23]. Furthermore, consistent with a part of our results, transmission among households in the UK performed higher secondary attack rates than those in communities, while relatively lower rates in larger households [24].

### Limitations

This study has some limitations. Firstly, the transmission cluster data used was subjected to any bias (e.g., recall bias) generated during the contact tracing process and thus it is plausible that some cases that are exposed to the clusters were missed. This imperfect case ascertainment may lead to an underestimation of the *R* value but an overestimation of the *k* value [25]. Secondly, disproportional attention to infectors who generated infectees or not may have resulted in that infectors generating infectees were more likely to be collected and reported. Besides, the transmission clusters included in our study occurred during the early stage of the COVID-19 epidemics. Finally, more combinations of different types of contact settings can be considered when some places are interconnected through ventilation. Given that the current epidemics are dominated by the SARS-CoV-2 Omicron variants, further study is warranted to assess the superspreading potential of the emerging variants in Japan and other regions to help with formulating control policy.

## Conclusion

In conclusion, the early COVID-19 epidemics in Japan demonstrated a significant potential of superspreading. Particularly, the school, health care facility and community had relatively higher potential of superspreading when compared to other contact settings. The different potential of superspreading in contact settings highlights the need to continuously monitor the transmissibility accompanied with the dispersion parameter, to timely identify high risk settings favoring the occurrence of SSE.

## Supplementary Information

**Additional file 1.** Technical details.

## Data Availability

The data used in this study were retrieved from [10], and the processed data and code were publicly available via https://github.com/plxzpnxZBD/SSE_inJP_firsthalf2020.

## References

[1] Vynnycky E, White R. An introduction to infectious disease modelling. OUP oxford. 2010.

[2] Chen PZ, Koopmans M, Fisman DN, Gu FX (2021). Understanding why superspreading drives the COVID-19 pandemic but not the H1N1 pandemic. Lancet Infect Dis.

[3] Frieden TR, Lee CT (2020). Identifying and interrupting superspreading events-implications for control of severe acute respiratory syndrome coronavirus 2. Emerg Infect Dis.

[4] Bauch CT (2021). Estimating the COVID-19 R number: a bargain with the devil?. Lancet Infect Dis.

[5] Galvani AP, May RM (2005). Dimensions of superspreading. Nature.

[6] Lloyd-Smith JO, Schreiber SJ, Kopp PE, Getz WM (2005). Superspreading and the effect of individual variation on disease emergence. Nature.

[7] Westbrook L. The dance club scene behind Hong Kong’s biggest coronavirus cluster. South China Morning Post 2020. https://www.scmp.com/news/hong-kong/society/article/3111507/dance-niche-hong-kong-social-scene-behind-citys-biggest.

[8] Kim D, Ali ST, Kim S, Jo J, Lim JS, Lee S, Ryu S (2022). Estimation of serial interval and reproduction number to quantify the transmissibility of SARS-CoV-2 omicron variant in South Korea. Viruses.

[9] Lewis D. *Superspreading drives the COVID pandemic - and could help to tame it*. Nature News. 2021. Retrieved August 1, 2022, from https://www.nature.com/articles/d41586-021-00460-x#:~:text=23%20February%202021-,Superspreading%20drives%20the%20COVID%20pandemic%20%E2%80%94%20and%20could%20help%20to%20tame,best%20to%20target%20control%20measures.&text=Dyani%20Lewis%20is%20a%20freelance%20science%20journalist%20in%20Melbourne%2C%20Australia. 10.1038/d41586-021-00460-x 33623168

[10] Furuse Y, Tsuchiya N, Miyahara R, Yasuda I, Sando E, Ko YK (2022). COVID-19 case-clusters and transmission chains in the communities in Japan. J Infect.

[11] Endo A, Abbott S, Kucharski AJ, Funk S (2020). Estimating the overdispersion in COVID-19 transmission using outbreak sizes outside China. Wellcome Open Res.

[12] Kucharski AJ, Althaus CL (2015). The role of superspreading in Middle East respiratory syndrome coronavirus (MERS-CoV) transmission. Eurosurveillance.

[13] Zhang S, Diao M, Yu W, Pei L, Lin Z, Chen D (2020). Estimation of the reproductive number of novel coronavirus (COVID-19) and the probable outbreak size on the Diamond Princess cruise ship: a data-driven analysis. Int J Infect Dis.

[14] Blumberg S, Funk S, Pulliam JR (2014). Detecting differential transmissibilities that affect the size of self-limited outbreaks. PLoS Pathog.

[15] Ko YK, Furuse Y, Ninomiya K, Otani K, Akaba H, Miyahara R (2022). Secondary transmission of SARS-CoV-2 during the first two waves in Japan: demographic characteristics and overdispersion. Int J Infect Dis.

[16] Adam DC, Wu P, Wong JY, Lau EH, Tsang TK, Cauchemez S (2020). Clustering and superspreading potential of SARS-CoV-2 infections in Hong Kong. Nat Med.

[17] Oshitani H, The Expert Members of The National COVID-19 Cluster Taskforce at The Ministry of Health, L. and W. Cluster-based approach to coronavirus disease 2019 (COVID-19) response in Japan, from February to April 2020. Jpn J Infect Dis. 2020. Retrieved August 1, 2022, from https://www.jstage.jst.go.jp/article/yoken/73/6/73_JJID.2020.363/_article. 10.7883/yoken.JJID.2020.363 32611985

[18] Cowling BJ, Ali ST, Ng TW, Tsang TK, Li JC, Fong MW (2020). Impact assessment of non-pharmaceutical interventions against coronavirus disease 2019 and influenza in Hong Kong: an observational study. The Lancet Public Health.

[19] Majra D, Benson J, Pitts J, Stebbing J. SARS-COV-2 (COVID-19) superspreader events. J Infect. 2020. Retrieved August 1, 2022, from https://www.sciencedirect.com/science/article/pii/S0163445320307179. 10.1016/j.jinf.2020.11.021 PMC7685932 33245943

[20] Luu MN, Alhady ST, Nguyen Tran MD, Truong LV, Qarawi A, Venkatesh U, Tiwari R, Rocha IC, Minh LH, Ravikulan R, Dumre SP (2022). Evaluation of risk factors associated with SARS-CoV-2 transmission. Curr Med Res Opin.

[21] Guo Z, Zhao S, Ryu S, Mok CKP, Hung CT, Chong KC, Yeoh EK. Superspreading potential of infection seeded by the SARS-CoV-2 Omicron BA. 1 variant in South Korea. J Infect. 2022. 10.1016/j.jinf.2022.05.041 PMC9158374 35659549

[22] Rocha IC, Pelayo MG, Rackimuthu S (2021). Kumbh mela religious gathering as a massive superspreading event: potential culprit for the exponential surge of COVID-19 cases in India. Am J Trop Med Hyg.

[23] Rocha IC, Pelayo MG, Sammatid C (2021). Traveling and celebrating during Songkran as super spreading events: a potential triggering factor of the surge of COVID-19 cases in Thailand. Int J Travel Med Global Health.

[24] Bernal JL, Panagiotopoulos N, Byers C, Vilaplana TG, Boddington N, Zhang XS, Charlett A, Elgohari S, Coughlan L, Whillock R, Logan S (2020). Transmission dynamics of COVID-19 in household and community settings in the United Kingdom. MedRxiv..

[25] Lloyd-Smith JO (2007). Maximum likelihood estimation of the negative binomial dispersion parameter for highly overdispersed data, with applications to infectious diseases. PLoS ONE.

