# Supplementary material for: Differences in the superspreading potentials of COVID-19 across contact settings
Source: BMC Infect Dis. 2022 Dec 12;22:936. doi: 10.1186/s12879-022-07928-9 (PMC9744370; doi:10.1186/s12879-022-07928-9)
Supplement: Supplementary file 1 — Additional file 1. Technical details. [file 12879_2022_7928_MOESM1_ESM.docx]

**Supplementary Information**

**Secondary case distribution in the context of superspreading**

Given the stochastic effect of the transmission events, the transmission dynamics can be modelled by a Poisson process, such that the number of secondary cases *Y* generated by each infector is described by a Poisson distribution a mean of $\lambda$ [1]. To characterize the heterogeneity in individual transmissibility, the $\lambda$ was assumed to follow a gamma distribution and thereby yield a Negative binomial secondary case distribution parameterized by the reproduction number (*R*) and a dispersion parameter (*k*) [2]. Therefore, the probability that an infector generates $y\left( \geq0 \right)$ secondary cases is given by

|  |  | (1) |
| --- | --- | --- |

where is the gamma function satisfying that . When a transmission cluster involves $x_{i}$ infectors who seeded a total of $y_{i}$ secondary cases, then the above function is adjusted as [4,5]:

|  | $r\left( x_{i},y_{i} \right)=Pr\left( Y=y_{i};R,k \right)=\frac{\Gamma\left( k*x_{i}+y_{i} \right)}{\Gamma\left( y_{i}+1 \right)\Gamma\left( k*x_{i} \right)}\left( \frac{k}{R+k} \right)^{k*x_{i}}\left( \frac{R}{R+k} \right)^{y_{i}}$ | (2) |
| --- | --- | --- |

Then, the likelihood function based on the dataset with totally $n$ transmission pairs is

|  |  | (3) |
| --- | --- | --- |

**Parameter estimation**

The Markov Chain Monte Carlo (MCMC) method was employed to jointly estimate the reproduction number *R* and dispersion parameter *k*. Metropolis-Hastings algorithm was adopted and the marginal posterior distributions were obtained from 110 000 MCMC iterations, among which the first 30 000 were discarded as burn-in. Uniform prior distributions were applied for *R* and *k*. The 95% credible intervals were drawn from the marginal posterior distributions. Trace plot and Gelman–Rubin diagnostic were used for checking the convergence of the MCMC [7]. All statistical analysis were performed in **R** version 4.1.1 (R Foundation for Statistical Computing).

**Measurements of superspreading potential**s

Armed with the estimated *R* and *k* values, we deduced the proportion of the most infectious cases responsible for 80% of total transmissions, which was formulated as per [9]:

|  |  | (4) |
| --- | --- | --- |

where represents the floor function and satisfies

|  |  | (5) |
| --- | --- | --- |

Followed by previous work [9], we defined the threshold of SSE for the COVID-19 as the 99^th^ percentile of the Poisson distribution of the basic reproduction number (*R*_0_). Given that a consensus *R*_0_ estimates were in a range of 2 to 3 [10], the threshold of SSE was determined to be 6 to 8. The threshold was assumed to be 6 in this study. Any transmission event that is directly seeded by a single infector would be counted as an SSE if the number of secondary cases exceeds the threshold (i.e., 6).

Then, the probability of observing an SSE seeded by a single infector is given by

| ${P_{s}=1-F(y;R,k)\vert}_{y=5\mathrm{or}7}$ | (6) |
| --- | --- |

Here, $F(.)$ is the cumulative probability function of equation (2).

Furthermore, based on the methods derived in [9,11], when we refocus on the final cluster size with the assumption that the offspring distribution are independently and identically distributed (iid) negative binomial distribution given by equation (2), the possibility mass function for the final size $s$ of th cluster caused by $x$ initial cases is given by:

|  | (7) |
| --- | --- |

Therefore, the probability of $x$ seed cases resulting in a cluster with size $s$ or more is .

**References**

1. Karlis, D., & Xekalaki, E. (2000). A simulation comparison of several procedures for testing the Poisson assumption. *Journal of the Royal Statistical Society: Series D (The Statistician)*, *49*(3), 355-382.
2. Lloyd-Smith, J. O., Schreiber, S. J., Kopp, P. E., & Getz, W. M. (2005). Superspreading and the effect of individual variation on disease emergence. *Nature*, *438*(7066), 355-359.
3. Furuse, Y., Tsuchiya, N., Miyahara, R., Yasuda, I., Sando, E., Ko, Y. K., ... & Oshitani, H. (2022). COVID-19 case-clusters and transmission chains in the communities in Japan. *Journal of Infection*, *84*(2), 248-288.
4. Adam, D. C., Wu, P., Wong, J. Y., Lau, E. H., Tsang, T. K., Cauchemez, S., ... & Cowling, B. J. (2020). Clustering and superspreading potential of SARS-CoV-2 infections in Hong Kong. *Nature Medicine*, *26*(11), 1714-1719.
5. Zhang, Y., Li, Y., Wang, L., Li, M., & Zhou, X. (2020). Evaluating transmission heterogeneity and super-spreading event of COVID-19 in a metropolis of China. *International journal of environmental research and public health*, *17*(10), 3705.
6. Hartig, F., Calabrese, J. M., Reineking, B., Wiegand, T., & Huth, A. (2011). Statistical inference for stochastic simulation models–theory and application. *Ecology letters*, *14*(8), 816-827.
7. Gelman, A., Carlin, J. B., Stern, H. S., & Rubin, D. B. (1995). *Bayesian data analysis*. Chapman and Hall/CRC.
8. Galvani, A. P., & May, R. M. (2005). Dimensions of superspreading. *Nature*, *438*(7066), 293-295.
9. Endo, A. (2020). Estimating the overdispersion in COVID-19 transmission using outbreak sizes outside China. *Wellcome open research*, *5*.
10. Zhang, S., Diao, M., Yu, W., Pei, L., Lin, Z., & Chen, D. (2020). Estimation of the reproductive number of novel coronavirus (COVID-19) and the probable outbreak size on the Diamond Princess cruise ship: A data-driven analysis. *International journal of infectious diseases*, *93*, 201-204.
11. Blumberg, S., Funk, S., & Pulliam, J. R. (2014). Detecting differential transmissibilities that affect the size of self-limited outbreaks. *PLoS pathogens*, *10*(10), e1004452.
